# Cyclin-dependent kinase 9 is required for the survival of adult *Drosophila melanogaster* glia

**DOI:** 10.1038/s41598-017-07179-8

**Published:** 2017-07-28

**Authors:** Lynette C. Foo

**Affiliations:** Institute of Molecular and Cell Biology, 61 Biopolis Dr, 138673 Singapore, Singapore

## Abstract

Neuronal and glial progenitor cells exist in the adult *Drosophila* brain. The primarily glial progenitor cells rely on a microRNA, *mir-31a*, to inhibit the expression of a predicted E3 ubiquitin ligase, CG16947. Erroneous inheritance of CG16947 by the progeny when the neural progenitor cell divides leads to death of the progeny, however how CG16947 achieves glial cell death is unknown. I have identified the interacting partner of CG16947 to be cdk9. I show that reduction of cdk9 expression in glia causes glial loss; highlighting the importance of cdk9 in mediating the survival of glia. Further, glial loss observed in *mir-31a* mutants was prevented with adult-specific expression of cdk9 in glia. I provide biochemical evidence that the binding of CG16947 to cdk9 causes its degradation. Taken together, this data shows that cdk9 plays a role in the survival of adult glia in the *Drosophila* brain. Thus, a fine balance exists between *mir-31a* and CG16947 expression in the progenitor cells that in turn regulates the levels of cdk9 in the progeny. This serves to allow the progenitor cells to regulate the number of glia in the adult brain.

## Introduction

The study of glial production in the adult has largely centred on aberrant glial division in injury or in disease states such as in the formation of glioblastoma. In mammals, the majority of glia are made post-embryonically and very little division is observed in the adult^1^. In *Drosophila melanogaster*, it has been shown that glia are still being produced in early adulthood by progenitor cells that persist into adulthood^2–4^. A subset of these predominantly glial progenitor cells express the microRNA, *mir-31a*, which inhibits the expression of a RING finger and CHY zinc finger domain containing 1, E3 ubiquitin ligase, CG16947. The mammalian homologue of CG16947 is rchy1. In *mir-31a* mutants, where the inhibition of CG16947 translation is lifted by the absence of *mir-31a*, there is excessive expression of CG16947 in the progenitor cells. This leads to aberrant inheritance of CG16947 by the glial progeny, leading to their death by apoptosis^4^. However, the mechanism by which CG16947 causes apoptotic glial cell death has not be elucidated.

Mammalian rchy1 has been shown to interact with proteins such as p53, p73 and cyclin dependent kinase 9 (cdk9)^5, 6^. To understand the mechanism of action of CG16947 in mediating apoptosis of glia in *Drosophila melanogaster*, I used a biochemical approach to elucidate the binding partner of CG16947. I found that CG16947 interacts with cdk9.

In mammals, cdk9 has been shown to bind, as part of a heterodimer complex with T cyclins or cyclin K, to and phosphorylate RNA polymerase II. This allows the stabilisation of the transcript of RNA being synthesised^7^. cdk9 itself has been shown to be important for cancer cell survival in primary human leukaemia^8^. Interestingly, it appears that *cdk9* expression is upregulated in immature mammalian microglia^5, 9^.

In this paper I show that reduction of cdk9 expression in glia in an otherwise wildtype background leads to loss of glia in 7d old adults. I also show that the expression of cdk9 in adult glia can prevent the glial loss observed in *mir-31a* mutants. Taken together, this data shows that cdk9 mediates the survival of glia in the adult *Drosophila* brain.

## Results

### Cdk9 is required for the survival of glia

CG16947 expression in a subset of progenitor cells in the adult brain is controlled by the microRNA, *mir-31a*. In *mir-31a* mutants, the absence of *mir-31a* leads to the overexpression of CG16947. This causes an increased inheritance of CG16947 by the glial progeny that in turn results in apoptotic cell death of the glial progeny. As a consequence, even though the flies eclose with the same number of glia, fewer glia in 7d old adult brains are observed^4^. Understanding how CG16947 causes glial cell death requires an understanding of its interacting partners. In mammals, rchy1 has been shown to interact with a number of proteins, in particular with p53^5, 6^.

**Figure 1:**
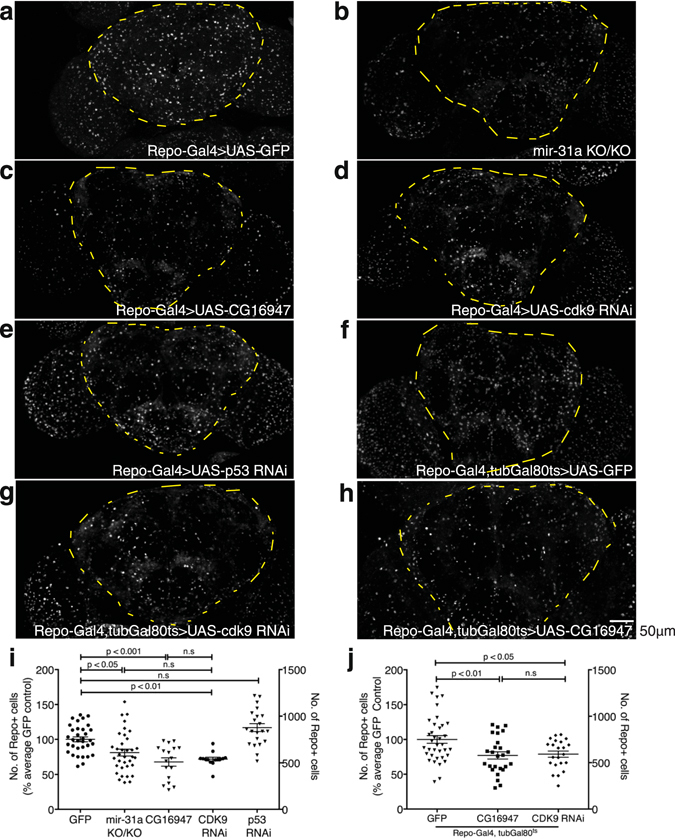
cdk9 is necessary for glial survival. (**a–h**) Representative images of 7d old adult brains immunostained with anti-Repo to label glia for Fig. 1i,j. The images are maximum projections of optical sections. The outlined area demarcates the region of the central brain where the number of glia were quantified. (**i**) Number of anti-Repo-expressing glia in the central brain of 7d old adult flies in *mir-31a* mutants (mir-31a KO/KO) and where *UAS-GFP*, *UAS-CG16947*, *UAS-cdk9* RNAi or *UAS-p53* RNAi lines were expressed in glia using *Repo-Gal4*. Glia were represented as a percentage of the control animals, *Repo-Gal4* > *UAS-GFP* (left axis). Raw number of Repo-expressing glia in the central brain of the genotypes (right axis). One-way ANOVA was used for statistical analysis and error bars represent SEM. (**j**) Number of anti-Repo-expressing glia in the central brain of 7d old adult flies. *UAS-GFP*, *UAS-CG16947* and *UAS-cdk9* RNAi expression was driven by *Repo-Gal4* under the control of tubulin Gal80^ts^ (tubGal80^ts^). Adult-only expression was achieved by rearing the flies at 18 °C then moving the flies to 29 °C to allow *Repo-Gal4* activity after eclosion. Data is represented as a percentage of the number of glia in the UAS-GFP control condition (left axis). Raw number of Repo-expressing glia in the central brain of the genotypes (right axis). One-way ANOVA was used for statistical analysis and error bars represent SEM.

To identify which of the potential interacting partners of CG16947 were responsible for mediating cell death in *Drosophila melanogaster*, I did an RNAi screen where I overexpressed candidate CG16947-interacting protein RNAi lines in glia with a pan-glial Gal4 driver, *Repo-Gal4* (*reversed polarity*), in an otherwise wildtype background. In the *mir-31a* mutants, flies eclose with the same number of glia but suffer significant glial loss by 7d post-eclosion^4^. Thus, I quantified the effect of the RNAi lines in the central brains of 7d post-eclosion adults. I hypothesised that if CG16947, being a predicted E3 ubiquitin ligase, was present at higher than normal levels in glia, it would cause ubiquitination and degradation of its interacting partner. Thus, knocking down the interacting partner of CG16947 should mimic the loss of glia observed in both *mir-31a* mutants and when CG16947 is overexpressed. As expected, overexpression of CG16947 in glia (*Repo-Gal4* > *UAS-CG16947*) reduces the number of glia in the brain compared to the control condition where GFP was overexpressed in accordance with what was previously observed^4^. Indeed, knocking down cdk9, led to a significant decrease in the number of glia in the adult brain at 7d post-eclosion. There was no significant difference in the number of glia between *mir-31a* mutants and in the genotype where cdk9 was knocked down. Knocking down of p53 in glia, in contrast, did not lead to a significant decrease in the number of glia in the brain. (Fig. 1a–e,i). This data shows that knocking down cdk9 but not p53 in glia leads to glial loss. Anti-Repo was used to immunostain and the number of glia in the central brain of 7d old adult animals were counted. A cartoon depicting the region quantified is in Supplemental Fig. 1a. The number of glia are represented as a percentage of the *Repo-Gal4* > *UAS-GFP* control animals (Fig. 1i, left axis) and the raw numbers depicted on the right axis (Fig. 1i).

To ensure that knocking down of cdk9 affected not the generation of glia in the adult brain, but their survival, the number of glia in the central brains of 1d old post-eclosion flies was also quantified. The number of glia generated upon eclosion was not significantly different between the *mir-31a* mutants, the otherwise wildtype controls of *Repo-Gal4* > *UAS-GFP* and the conditions were *UAS-CG16947* and *UAS-cdk9* RNAi was overexpressed at this age (Supplemental Fig. 1b–f). This demonstrates that knocking down of cdk9 in glia is detrimental not to gliogenesis, but to the survival of glia in the adult, just as what was shown in Foo *et al*. 2017 where fewer glia were seen in 7d old *mir-31a* mutant adults, but no difference was observed in 2d old animals when compared to age-matched control animals^4^.

To ensure that the effect of cdk9 affected only adult glia, I used a temperature-sensitive form of Gal80 (tubGal80^ts^) to control the expression of *Repo-Gal4* such that cdk9 was only knocked down when adult flies were moved from 18 °C, in which they were reared, to the permissive temperature of 29 °C, upon eclosion. This allowed the knocking down of cdk9 exclusively in adult glia. As a control, *Repo-Gal4, tubGal80* ^*ts*^ > *UAS-GFP* flies were reared entirely at 18 °C for 7d post-eclosion and their brains examined for aberrant expression of GFP. It was observed that there was no aberrant expression of GFP in these flies, unless they were moved to 29 °C for 1 day (permissive temperature) (Supplemental Fig. 1g,h). This demonstrates that the tubGal80^ts^ was effective at suppressing the activity of *Repo-Gal4*.

The flies were dissected 7d after eclosion. The adult-specific depletion of cdk9 was sufficient to reduce the number of glia in the brain compared to control animals where GFP was overexpressed instead. The number of glia in the brain observed when cdk9 was knocked down in adult glia was comparable to the number of glia observed when CG16947 was overexpressed in adult glia alone (Fig. 1f–h,j).

**Figure 2:**
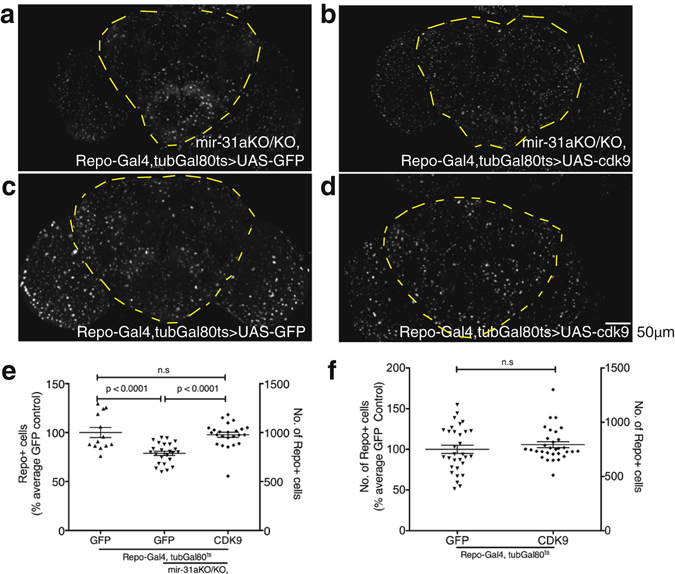
cdk9 is necessary for adult glial survival. (**a–d**) Representative images of 7d old adult brains immunostained with anti-Repo to label glia for Fig. 2e,f. The images are maximum projections of optical sections. The outlined area demarcates the region of the central brain where the number of glia were quantified. (**e**) Number of anti-Repo-expressing cells in the central brain of 7d old *mir-31a* mutants (mir-31a KO/KO) when either *UAS-GFP* or *UAS-cdk9* was expressed only in adult glia with *Repo-Gal4* under the control of tubGal80^ts^. *Repo-Gal4* > *UAS-GFP* represent otherwise wildtype control animals to demonstrate the effectiveness of CDK9 at restoring the wildtype levels of glia in the adult brain. Data is represented as a percentage of the number of glia in the *UAS-GFP* control condition (left axis). Raw number of Repo-expressing glia in the central brain of the genotypes (right axis). One-way ANOVA was used. Error bars represent SEM. (**f**) Number of anti-Repo-expressing cells in the central brain of 7d old adults expressing either *UAS-GFP* or *UAS-cdk9* only in glia with *Repo-Gal4* under the control of Gal80^ts^. Data is represented as a percentage of the number of glia in the *UAS-GF*P control condition (left axis). Raw number of Repo-expressing glia in the central brain of the genotypes (right axis). Unpaired Student’s t-test was used. Error bars represent SEM.

*mir-31a* mutants have fewer glia in the 7d old adult brains due to aberrant inheritance of CG16947 by the progeny^4^. If cdk9 is the interacting partner of CG16947 and that its depletion is the cause of glial cell death in the adult, then overexpressing cdk9 only in the *mir-31a* mutant background should be sufficient to prevent glial loss. As the flies failed to eclose when cdk9 was constitutively overexpressed with *Repo-Gal4*, I used tubGal80^ts^ to overexpress cdk9 in adult glia only. Indeed, overexpression of cdk9 in adult glia using *Repo-Gal4* under the control of tubGal80^ts^ was able to prevent the glial loss observed in *mir-31a* mutants. When GFP was expressed instead, glial loss persisted (Fig. 2a,b,e). Cdk9 was able to restore the number of glia in *mir-31a* mutant brains to levels observed in an otherwise wildtype control animal, where *Repo-Gal4* was used to drive the expression of *UAS-GFP*. This data suggests that cdk9 acts downstream of *mir-31a* in the progeny to control glial cell survival.

In mammals, more than 50% of the oligodendrocytes generated die by apoptosis during development. Oligodendrocytes are dependent on the presence of axons for survival^10^ and the oligodendrocytes that do not receive sufficient trophic support will die by apoptosis^11^. It is in this way that it is thought oligodendrocytes are matched to the axons that they myelinate. The regulation of glia number in the brain by contact with another cell type is also observed in regulation of astrocyte number in the rodent brain. Majority of astrocytes contact blood vessels and astrocytes are reliant on trophic support from the vasculature. Failure to make contact with the vasculature leads to death by apoptosis^12^. If a similar situation where more glia are generated in *Drosophila* adult brains than are required, then artificially overexpressing cdk9 in adult glia alone would lead to an increase in the number of glia observed. In contrast to what is observed in the developing mammalian brain, when cdk9 was overexpressed with *Repo-Gal4* under the control of tubGal80^ts^ such that cdk9 was only overexpressed when the newly eclosed flies were moved to 29 °C for 7d before dissection, I found that there was no increase in the number of glia observed compared to control animals where GFP was overexpressed (Fig. 2c,d,f). This suggests that overexpression of cdk9 alone is not sufficient to increase the number of glia in the brain in an otherwise wildtype background.

### cdk9 is a direct interacting partner of CG16947

I used biochemistry to determine if cdk9 is a direct interacting partner of CG16947. I overexpressed either GFP or CG16947 with *Repo-Gal4* and collected and processed whole flies for co-immunoprecipitation with anti-CG16947, that had previously been shown to recognise *Drosophila* CG16947^4^. The flies were processed 1d post-eclosion as it had previously been shown that the *mir-31a* mutant animals eclose with the same number of glia but lose their glia by 7d of age. Thus, processing the animals at 1d would ensure that the glia had not yet undergone apoptosis.

**Figure 3:**
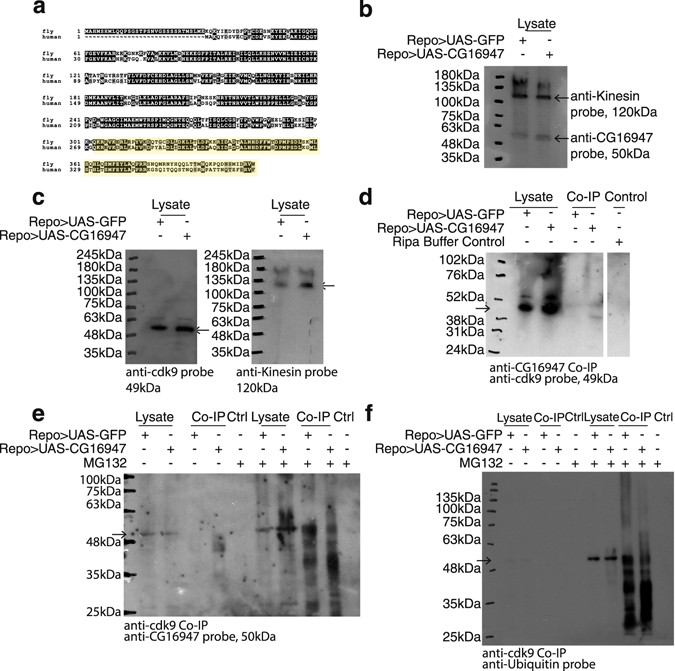
cdk9 interacts directly with CG16947. (**a**) Alignment of the *Drosophila melanogaster* (fly) and human protein sequences of cdk9. Highlighted portion denotes region that the commercial anti-cdk9 antibody was generated against human cdk9 protein. (**b**) CG16947 (50 kDa) was detected in the lysates from *Repo-Gal4* > *UAS-GFP* (Repo > UAS-GFP) and *Repo-Gal4* > *UAS-CG16947* (Repo > UAS-CG16947) used for co-immunoprecipitation in 3d, e, f and Supplemental Fig. 2. Kinesin (120 kDa) was used as a loading control to ensure that similar levels of protein were loaded from both genotypes. (**c**) cdk9 (49 kDa) was detected in the lysates from *Repo-Gal4* > *UAS-GFP* (Repo > UAS-GFP) and *Repo-Gal4* > *UAS-CG16947* (Repo > UAS-CG16947) used for co-immunoprecipitation in 3d, e, f and Supplemental Fig S2. Kinesin (120 kDa) was used as a loading control to ensure that similar levels of protein were loaded from both genotypes. (**d**) Western blot of anti-CG16947 co-immunoprecipitation. Flies were of either *Repo-Gal4* > *UAS-GFP* (Repo > UAS-GFP) or *Repo* > *UAS-CG16947* (Repo > UAS-CG16947) genotype. Blot was probed with anti-cdk9. Lysate refers to the input for the co-immunoprecipitation. A control condition where the beads were incubated with the anti-CG16947 antibody in the absence of lysate was done to distinguish anti-CG16947 antibody elution from the beads versus the CG16947 protein itself. A 49 kDa cdk9 band was detected in the lysates and the without DTT elution of the anti-rchy1 pulldown in both *Repo-Gal4* > *UAS-GFP* (Repo > UAS-GFP) and *Repo-Gal4* > *UAS-CG16947* (Repo > UAS-CG16947) conditions. (**e**) Co-immunoprecipitation with anti-cdk9. Anti-cdk9 pulled down CG16947 protein (50 kDa). An increase in the number and intensity of the bands recognised by anti-CDK9 in the MG132-treated samples compared to when the lysate and co-immunoprecipitation was done without MG132 suggest that CG16947 was actively degraded via the proteasome *in vitro*. The multiple bands observed below the expected size for CG16947 likely represent degraded CG16947, which can be observed when the proteasome is inhibited *in vitro*. (**f**) Co-immunoprecipitation with anti-cdk9. anti-cdk9 pulled down cdk9 protein that was ubiquitinated as shown by the bands above the expected size for cdk9, 49 kDa and degraded, as shown by the bands below 49 kDa. An increase in the number and intensity of the bands recognised by anti-ubiquitin in the MG132-treated samples demonstrated that cdk9 was actively ubiquitinated and degraded via the proteasome *in vitro*. A higher exposure of the blot is shown in Supplemental Fig. 2 where ubiquitinated cdk9 and its degraded products can be detected in the lysates without MG132.

In comparing the protein sequences of *Drosophila* and human cdk9 proteins, I found that there was considerable similarity between the two. I used an antibody that recognises the amino acid residues from position 271 to 372 of the human protein (shaded in yellow, Fig. 3a). As shown in the box shade plot in Fig. 3a, there are long stretches of homology between the two species within this region of the protein.

First I verified that I was able to detect both CG16947 (50 kDa) and cdk9 (49 kDa) in the lysates. CG16947 and cdk9 was detectable in lysates from both genotypes (Fig. 3b,c).

*Drosophila* cdk9 is estimated to be 49 kDa, which is close to the molecular weight of the heavy chain of the antibody. As such, I incubated the antibody with beads in the absence of any lysate as a control. A 49 kDa band representing cdk9 is observed in the lysate alone in both *Repo-Gal4* > *UAS-GFP* and *Repo-Gal4* > *UAS-CG16947* conditions. As shown in Fig. 3d, the elution was sufficient to observe the 49 kDa cdk9 band but insufficient to elute the antibody itself from the beads as shown in the RIPA buffer control sample lane. I observed a 49 kDa band in both the *Repo-Gal4* > *UAS-GFP* and *Repo-Gal4* > *UAS-CG16947* co-immunoprecipitation conditions. These results suggest that cdk9 is a direct interacting partner of CG16947.

To ensure that there is in fact a direct interaction between, cdk9 and CG16947, I co-immunoprecipitated the lysates with anti-cdk9 and probed for anti-CG16947 in the presence or absence of MG132, a proteasome inhibitor. Lysis and co-immunoprecipitation in the presence of MG132 will slow down the *in vitro* degradation of proteins that have been ubiquitinated and are slated for degradation by the proteasome. In accordance with Fig. 3b, CG16947 is detected in the lysates of both *Repo-Gal4* > *UAS-GFP* and *Repo-Gal4* > *UAS-CG16947*. The protein is absent in the anti-cdk9 pulldown lanes for both genotypes and in the control condition where anti-cdk9 was incubated with RIPA buffer alone. However, in the condition where the co-immunoprecipitation was performed in the presence of MG132, a 50 kDa band is observed in both the *Repo-Gal4* > *UAS-GFP* and *Repo-Gal4* > *UAS-CG16947* co-immunoprecipitates. This suggests that it is likely that CG16947 is also degraded rapidly by the proteasome *in vitro* once it has bound to cdk9 protein. Accordingly, multiple bands beneath the expected size of 50 kDa for CG16947 were detected, likely representing the antibody recognising degraded CG16947.

In order to determine if the interaction between cdk9 and CG16947 leads to its ubiquitination and subsequent degradation, I co-immunoprecipitated with anti-cdk9 and probed for anti-ubiquitin. Ubiquitination was observed in the lysate of both *Repo-Gal4* > *UAS-GFP* and *Repo-Gal4* > *UAS-CG16947* conditions (Supplemental Fig. 2, higher exposure blot). Anti-ubiquitin recognised proteins both larger, indicating ubiquitination, and smaller, indicating degradation, than the predicted size for cdk9, 49 kDa. The laddering that is observed in both the *Repo-Gal4* > *UAS-GFP* and *Repo-Gal4* > *UAS-CG16947* co-immunoprecipitation conditions are characteristic of ubiquitination and degradation of a protein (Supplemental Fig. 2, higher exposure blot). This data suggests that cdk9 is being actively degraded following ubiquitination.

To confirm that the ubiquitin-proteasome system is indeed involved in the degradation of cdk9, I conducted the protein extraction and co-immunoprecipitation of cdk9 in the presence of the proteasome inhibitor MG132 and probed for anti-ubiquitin. MG132-treated lysates showed an accumulation of ubiquitinated cdk9 as shown by the increased intensity and number of bands recognised by anti-ubiquitin compared to when the co-immunoprecipitation was conducted with lysates without MG132 (Fig. 3f, Supplemental Fig. 2).

## Discussion

CG16947 is expressed in the progenitor cells and *mir-31a* serves to limit its expression in the progenitor cells. When this interaction between the microRNA and the CG16947 transcript is abolished, CG16947 is aberrantly translated at higher levels, leading to increased inheritance by the progeny^4^.

In this paper, I show that depletion of cdk9 alone in adult glia can reduce the number of glia in the adult brain. Thus, cdk9 is necessary for adult glial survival. Additionally, I show that in the condition where there are fewer glia in the brain; as in the *mir-31a* mutants, the adult-specific overexpression of cdk9 in glia prevents glial loss. This shows that it is loss of cdk9 in adult glia that is the likely reason for the glial loss observed in *mir-31a* mutants.

Using co-immunoprecipitation, I provided evidence that CG16947 binds to and interacts directly with cdk9 leading to its degradation. Taken together, the genetic results and the biochemistry suggest that the subsequent ubiquitination and degradation of cdk9 due to excessive inheritance of CG16947 by the progeny in *mir-31a* mutants, is the likely cause for glial cell death in these mutants. This study does not exclude the possibility that cdk9 is degraded by other mechanisms, but do show that cdk9 is necessary for glial cell survival. The mechanism by which cdk9 mediates glial cell survival is beyond the scope of this paper. However, given the role of cdk9 in controlling RNA-polymerase II-mediated transcription, it is possible that cdk9 works by facilitating the transcription of pro-survival genes^7^.

As I did not observe an increase in glial number in the brain when cdk9 was overexpressed in adult glia, it suggests that cdk9 is not sufficient to generate more glial cells unless in aberrant conditions where glia number is reduced, as is in the case of the *mir-31a* mutants^4^.

Knocking down of CG16947 in progenitor cells did not lead to an increase in glia numbers in the brain^4^. Thus, it suggests that glial numbers are very tightly regulated in the adult brain. The rational being that since high levels of CG16947 inherited by glia from the progenitor cells is detrimental to the survival of glia then, in the wildtype setting, if more glia than necessary are made, then knocking down of CG16947 in progenitor cells, would lead to increased survival of glia and more glia observed. But as more glia is not observed in either setting, when CG16947 is knocked down or cdk9 is overexpressed, it suggests that the number of glia that are made is very tightly regulated and no more than necessary glia are made by the progenitors in the adult central brain of flies.

In mammals, there are several examples of the number of glia in the mature brain being tightly regulated by contact with a target. For instance, mammalian astrocytes are thought to be matched to blood vessels, mediated by the limited secretion of trophic factors from the vasculature^12^. A similar reliance of oligodendrocytes on their target axons for survival has also been observed^11^. Here I show that the number of glia can be in part, regulated from the progenitor cell itself, through controlled expression of CG16947, rather than reliance on the target of the glial cell. These results do not exclude the possibility that glial number in the *Drosophila* adult brain is regulated by another mechanism.

The mechanism by which *mir-31a* expression in the progenitor cell is regulated and in turn CG16947 expression is regulated, has yet to be uncovered, however, I provide evidence that misregulation of this fine balance between *mir-31a* with CG16947 in the progenitor cells and subsequent excessive degradation of cdk9 in the progeny can alter the number of glia in the adult *Drosophila* brain. Thus providing evidence for a mechanism by which the progenitor cell itself can regulate the number of progeny that are generated. This study does not exclude the possibility that cdk9 can be regulated in a *mir-31a*-CG16947-independent manner, but illustrates one way in which cdk9 expression can be regulated in glia.

## Methods

### Fly stocks

Flies were reared at 25 °C or 18 °C on standard fly media. The *mir-31a* mutant used was generated as described in ref. 13. *Repo-Gal4*, *UAS-CD8-GFP, UAS-CDK9 RNAi* (#41932) lines were obtained from Bloomington Drosophila Stock Centre. *UAS-CDK9* was obtained from FlyORF (#F000739). *UAS-CG16947* (#{XP}d01131) was obtained from the Harvard Exelixis Collection stocks.

### Immunostaining

Fly brains were dissected in cold PBS with 0.1% Tween-20 (PBST) at the ages stated in the figure legends. The flies were fixed for 20 mins in PBS with 0.1% Tween-20 with 4% paraformaldehyde. The brains were washed with PBST once before blocking with 3% bovine serum albumin for 30 mins. Thereafter, anti-Repo (Developmental Studies Hybridoma Bank, IA, USA, 1:20, #8D12) diluted in antibody buffer (150 mM NaCl, 50 mM Tris Base, 100mM L-lysine, 1% BSA, 0.04% azide, PH7.4) was incubated 36–48 h at 4 °C. After 4 washes with 1 ml of PBST, secondary antibody diluted in antibody buffer was incubated overnight at 4 °C. Alexa Fluor anti-mouse 488, 555 and 633 highly cross-adsorbed antibodies were used at 1:1000 (Thermo Fisher, MA, USA). The brains were then washed in 1 ml of PBST 4 times and then mounted in mounting media (0.2 M Tris-HCl, pH 8.5, 2.5% -*n*-propyl gallate and 90% glycerol)^14^. A Zeiss confocal Imager M2 was used to image the samples. Image J(NIH) was used with the ITCN plugin (Centre for Bio-image Informatics at UC Santa Barbara) for the counting of the number of glial cells in the central brain. The area counted is depicted by the yellow markings in Fig. 1. To demarcate the central brain, an outline was drawn around the central brain, excluding the flanking optic lobes, in Image J. The number of glia in the region demarcated was then counted using the ITCN plugin with the same settings for each image.

### Co-Immunoprecipitation

Approximately 1000 whole flies of each genotype were crushed in 200 µl of RIPA buffer (50 mM Tris-HCl pH 7.4, 150 mM NaCl, 1 mM EDTA, 1% Triton-X, 0.1% SDS) with 1x EDTA-free protease inhibitor cocktail (Roche LifeScience) on ice with a pestle mixer (Argos Technologies). The concentration of protein was determined with a Bradford Protein Assay (Bio-Rad). 2.5 mg of protein was incubated together with 2 µg of rabbit anti-CG16947 (Lifespan Biosciences, LS-B13598^4^), or 2 µg of mouse anti-cdk9 (Novus Biologicals, H00001025-M07) overnight on a tube rotator at 4 °C. The anti-cdk9 antibody used recognised a 49 kDa band, which corresponds to the predicted size for *Drosophila melanogaster* cdk9.

MG132 (final concentration 10 µM, Sigma Aldrich C2211) was added in the RIPA lysis buffer when the flies were crushed and during the entire procedure of co-immunoprecipitation.

nProtein A Sepharose 4 Fast Flow beads (GE Healthcare Life Sciences) were used. The beads were washed 1x in dPBS, then blocked with 0.2% BSA (diluted in dPBS) for 30 mins at room temperature. The beads were washed 2 × 1 ml dPBS, 5 min washes with tube inversion at room temperature and then washed 1 × 1 ml ice cold dPBS immediately before addition of the lysate + antibody on the second day.

The beads were spun at 2,000 g for 5 mins to pellet the beads between washes. The lysate + antibody was allowed to complex at 4 °C to the sepharose beads on a tube inverter over the course of 4 h. Subsequently, the beads were spun down at 2,000 g for 15 mins. The supernatant was discarded and the beads washed with cold dPBS 4 × 1 ml with tube inversion, 10 mins per wash. The beads were spun down at 2,000 g for 5 mins between each wash. After the final wash, the beads were pelleted at 2,000 g for 15 mins.

The elution was done by addition of 50 µl of 2xSDS loading buffer without DTT for 10 mins at 50 °C. The beads were then pelleted at 2,000 g for 5 mins and the supernatant transferred to a new tube. DTT was then added to a final concentration of 100 mM.

### Western blot

Lysate (input, 20 µg per lane for Fig. 3b,c; 10 µg per lane for Fig. 3d, 5 µg for Fig. 3e,f Supplemental Fig. 2) with 1x loading buffer (2 g SDS, 1 mg bromophenol blue, 0.78 ml glycerol, 1.2 ml of 0.5 M Tris, 2.1 ml water and 0.93 g DTT), and the co-immunoprecipitation elutions, were all boiled for 5 mins at 95 °C. 10 µl of eluate from the beads were run for Fig. 3d and 5 µl of eluate from the beads were run for Fig. 3e,f and Supplemental Fig. 2. Samples for the co-immunoprecipitation with anti-CG16947 were run on 10% polyacrylamide gels or 4–15% Bio-Rad gradient gels. Samples for the co-immunoprecipitation with anti-cdk9 were run on 10% polyacrylamide gels. Gels were run till the dye front ran out of the gel at the bottom, approximately 1 h at 150 V. Polyvinylidene Difluoride (PVDF) membranes were activated for 10 s with 100% methanol and used for the transfer. The transfer was done with 20% methanol in the transfer buffer (1X transfer buffer contained 9 g glycine, 1.9 g Tris-Base) for 45 mins at a steady voltage set at 10 V using the Bio-Rad Trans-Blot Semi-Dry Electrophoretic Transfer Cell (Bio-Rad). Membranes were blocked for 30 mins in 5% non-fat milk powder diluted in PBS containing 0.1% Triton-X (PBST-w). The membranes was quickly washed 2x with PBST-w before being incubated with anti-CG16947 (1:2000 dliuted in antibody buffer: 150 mM NaCl, 50 mM Tris Base, 100mM L-lysine, 1% BSA, 0.04% azide, PH7.4, Sigma, HPA030339), anti-cdk9 (1:750 diluted in antibody buffer, Novus Biologicals, H00001025-M07) or anti-ubiquitin (1:10,000 diluted in antibody buffer, Cell Signaling, 3933 S) overnight on a gentle rocker at 4 °C. Rabbit anti-kinesin (1:10,000, Cytoskeleton, AKIN01) was used as a loading control for the lysates. The primary antibodies were washed off and the blots washed in PBST-w 4x at room temperature the following day. Secondary antibodies were incubated at 1:10,000 in 5% milk diluted in PBST-w for 2 h at room temperature. The blots were subsequently washed 4x with PBST-w at room temperature. The blots were exposed with Amersham ECL Prime Western Blotting Detection Reagent (GE Healthcare Life Sciences).

### Statistics

Statistics were calculated with Prism 6 software (Graphpad, CA, USA). Unpaired Student’s t-test and One-way ANOVA with post-hoc Tukey analysis were the statistical tests used. Number of samples for each genotype used in Fig. 2, Supplemental Fig. 1 is represented as scatter plots in Fig. 2 and Supplemental Fig. 1 and the numerical numbers in Table 1.

**Table 1:** Table depicting number of samples, mean and SEM of all genotypes tested.

Figure	Genotype	No. of Samples	Mean	SEM	Viability
1i	mir-31a KO/KO	35	587.8	35.64	Viable
	Repo-Gal4 > UAS-GFP	17	722	25.38	Viable
	Repo-Gal4 > UAS-CG16947	33	490.3	43.47	Viable
	Repo-Gal4 > UAS-cdk9 RNAi	14	569.6	60.84	Viable
	Repo-Gal4 > UAS-p35 RNAi	21	843.7	41.2	Viable
1j	Repo-Gal4,tubGal80^ts^ > UAS-GFP	36	682.3	38.04	Viable
	Repo-Gal4, tubGal80^ts^ > UAS-CG16947	26	525.5	35.76	Viable
	Repo-Gal4, tubGal80^ts^ > UAS-CDK9 RNAi	22	538.3	30.65	Viable
2e	Repo-Gal4, tubGal80^ts^ > UAS-GFP	13	994.7	63.7	Viable
	Repo-Gal4,Gal80^ts^ > GFP, mir-31a KO/KO	24	746.2	20.43	Viable
	Repo-Gal4, tubGal80^ts^ > CDK9, mir-31a KO/KO	24	925	24.36	Viable
2f	Repo-Gal4, tubGal80^ts^ > GFP	31	780.7	39.12	Viable
	Repo-Gal4, tubGal80^ts^ > CDK9	31	825.3	28.26	Viable
Supp Fig. 1f	Repo-Gal4 > UAS-GFP 1d	15	642.6	47.8	Viable
	mir-31a KO/KO 1d	14	503.4	19.7	Viable
	Repo-Gal4 > UAS-CG16947 1d	11	602.8	51.68	Viable
	Repo-Gal4 > UAS-cdk9 RNAi 1d	10	604.9	57.66	Viable

### Box-shade plot analysis

http://www.ch.embnet.org/software/BOX_form.html was used to compare the aligned protein sequences of human and *Drosophila melanogaster* cdk9.

## Electronic supplementary material

Supplementary File
