# Supplementary material for: Cyclin-dependent kinase 9 is required for the survival of adult Drosophila melanogaster glia
Source: Sci Rep. 2017 Jul 28;7:6796. doi: 10.1038/s41598-017-07179-8 (PMC5533735; doi:10.1038/s41598-017-07179-8)
Supplement: Supplementary file 1 — Supplementary File [file 41598_2017_7179_MOESM1_ESM.pdf]

Supplementary Information

Cyclin-dependent kinase 9 is required for the survival of adult *Drosophila melanogaster* glia

Lynette C. Foo

Institute of Molecular and Cell Biology 61 Biopolis Dr, 138673 Singapore
Correspondence can be addressed to lynettefoo@gmail.com

Supplemental Figure Legends

Supplemental Figure Legend 1 Adult glia are reliant on cdk9 for survival

(**a**) Cartoon depiction of a *Drosophila* brain. Yellow shading indicates the central brain area that was quantified for all experiments.
(**b-e**) Representative images for Supplemental Fig 1f.
(**f**) *Repo-Gal4* was used to drive the expression of *UAS-GFP, UAS-CG16947* or *UAS-cdk* RNAi. *mir-31a* mutant animals were included as a comparison (mir-31a KO/KO). Number of anti-Repo-expressing cells in the central brain of 1 day post-eclosion animals represented as a percentage average of the otherwise wildtype control animals of *Repo-Gal4>UAS-GFP* (left axis). Raw number of anti-Repo-expressing cells in the central brain of 1 day post-eclosion animals (right axis). One-way ANOVA was used and scale bars represent SEM.
(**g**) *Repo-Gal4, tubGal80^{ts}>UAS-GFP* flies were reared entirely at 18°C and the brains of 7d old adult flies dissected to assess the ability of Gal80ts to suppress the activity of *Repo-Gal4.* DAPI was used to stain nuclei.
(**h**) *Repo-Gal4, tubGal80^{ts}>UAS-GFP* were reared entirely at 18°C until 7d post-eclosion whereupon they were moved to the permissive temperature of 29°C to inactivate the tubGal80^{ts} and permit the activity of *Repo-Gal4* and thus the expression of *UAS-GFP.* DAPI was used to stain nuclei.

Supplemental Figure Legend 2 cdk9 is degraded by the proteasome following ubiquitination

Co-immunoprecipitation with anti-cdk9 and probing with anti-ubiquitin antibody. Higher exposure of Fig 3f.

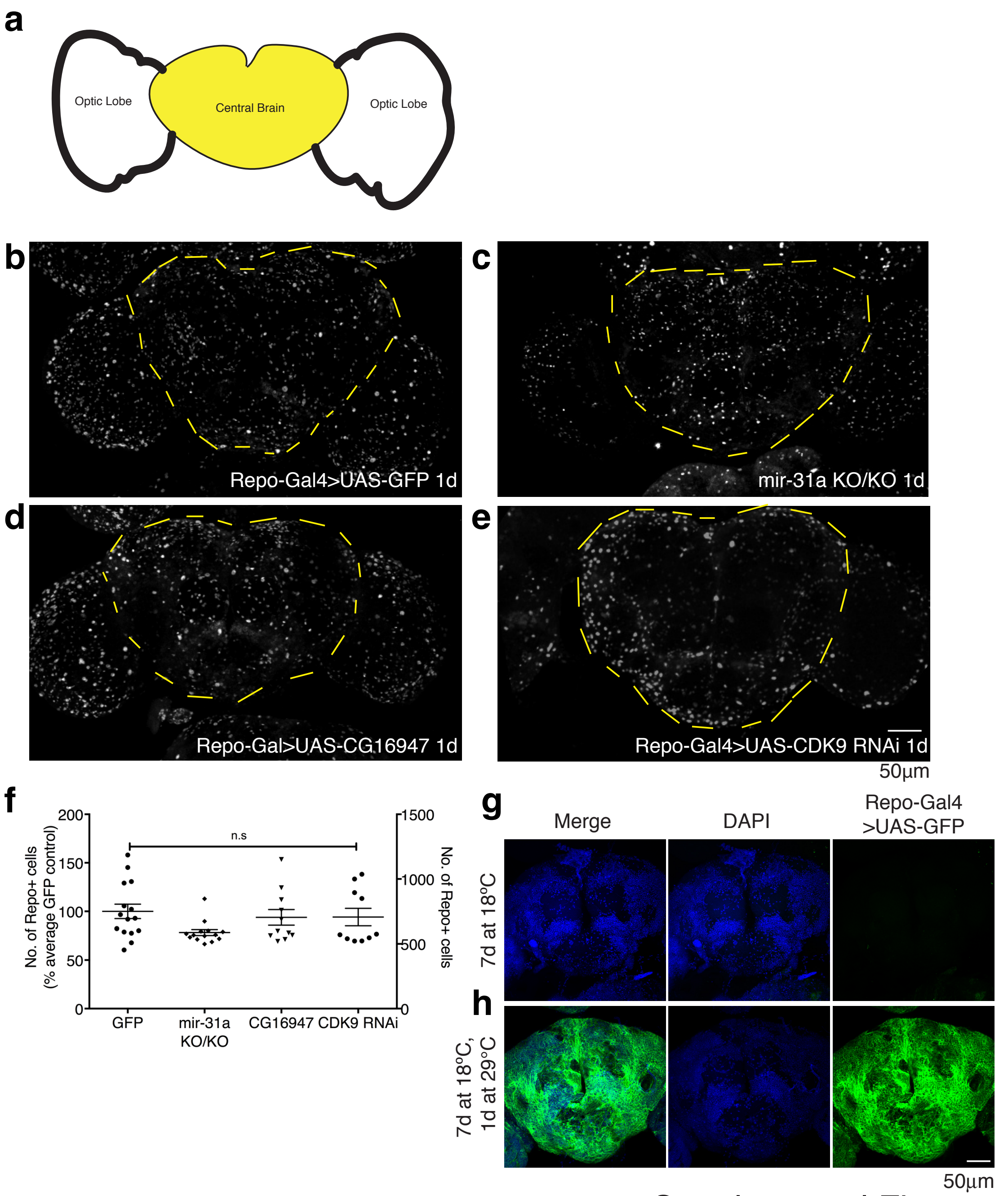

Supplemental Figure 1

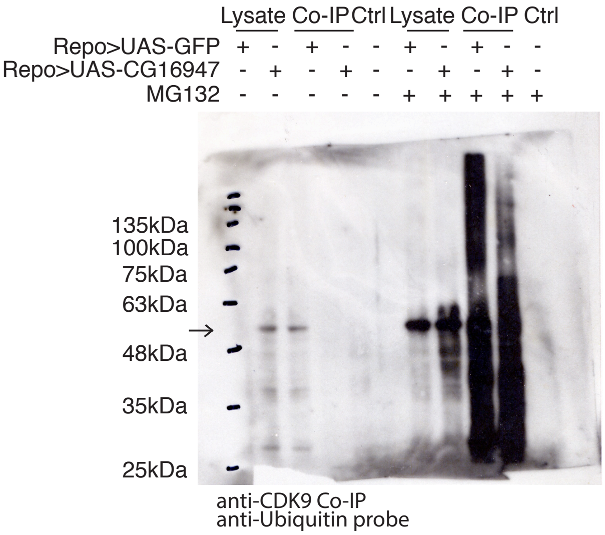

Supplemental Figure 2
